# 3-Acetyl-6-chloro-1-ethyl-4-phenyl­quinolin-2(1*H*)-one

**DOI:** 10.1107/S1600536809024830

**Published:** 2009-07-08

**Authors:** R. Subashini, Venkatesha R. Hathwar, T. Maiyalagan, G. Ganesh Kumar Reddy, F. Nawaz Khan

**Affiliations:** a Chemistry Division, School of Science and Humanities, VIT University, Vellore 632 014, Tamil Nadu, India; b Solid State and Structural Chemistry Unit, Indian Institute of Science, Bangalore 560 012, Karnataka, India

## Abstract

In the title compound, C_19_H_16_ClNO_2_, the dihedral angle between the plane of the phenyl substituent and 3-acetyl­quinoline unit is 75.44 (5)°. The crystal structure is stabilized by inter­molecular C—H⋯O hydrogen bonds

## Related literature

For general background to isoquinolines, see: Broadhurst *et al.* (2001 ▶); Behrens (1999 ▶); Broadhurst (1991 ▶); Chao *et al.* (1999 ▶); Cobet & Luckner (1971 ▶); Kametani (1968 ▶); Lamberton & Price (1953 ▶); Majumdar & Mukhopadhyay (2003 ▶); Nayar *et al.* (1971 ▶); Storer *et al.* (1973 ▶); Yong *et al.* (2001 ▶). For related crystal structures, see: Yang *et al.* (2008 ▶); Choudhury & Guru Row (2006 ▶); Choudhury *et al.* (2002 ▶); Hathwar *et al.* (2008 ▶); Cho *et al.* (2002 ▶); Manivel *et al.* (2009 ▶).
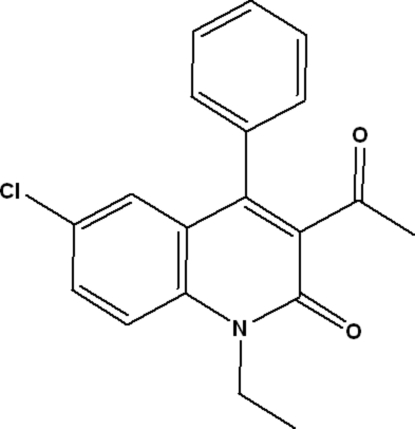


## Experimental

### 

#### Crystal data

C_19_H_16_ClNO_2_
*M* *_r_* = 325.78
Monoclinic, 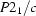

*a* = 9.6480 (8) Å
*b* = 17.5756 (11) Å
*c* = 9.9694 (7) Å
β = 103.245 (8)°
*V* = 1645.5 (2) Å^3^
*Z* = 4
Mo *K*α radiation
μ = 0.24 mm^−1^
*T* = 290 K
0.21 × 0.16 × 0.15 mm

#### Data collection

Oxford Xcalibur Eos(Nova) CCD detector diffractometer
Absorption correction: multi-scan (*CrysAlisPro RED*; Oxford Diffraction, 2009 ▶) *T*_min_ = 0.925, *T*_max_ = 0.965
21440 measured reflections
3061 independent reflections
1928 reflections with *I* > 2σ(*I*)
*R*_int_ = 0.052

#### Refinement

*R*[*F* ^2^ > 2σ(*F* ^2^)] = 0.040
*wR*(*F* ^2^) = 0.106
*S* = 0.95
3061 reflections
210 parameters
H-atom parameters constrained
Δρ_max_ = 0.16 e Å^−3^
Δρ_min_ = −0.27 e Å^−3^

### 

Data collection: *CrysAlisPro CCD* (Oxford Diffraction, 2009 ▶); cell refinement: *CrysAlisPro CCD*; data reduction: *CrysAlisPro RED* (Oxford Diffraction, 2009 ▶); program(s) used to solve structure: *SHELXS97* (Sheldrick, 2008 ▶); program(s) used to refine structure: *SHELXL97* (Sheldrick, 2008 ▶); molecular graphics: *CAMERON* (Watkin *et al.*, 1993 ▶); software used to prepare material for publication: *WinGX* (Farrugia, 1999 ▶).

## Supplementary Material

Crystal structure: contains datablocks global, I. DOI: 10.1107/S1600536809024830/bt2983sup1.cif

Structure factors: contains datablocks I. DOI: 10.1107/S1600536809024830/bt2983Isup2.hkl

Additional supplementary materials:  crystallographic information; 3D view; checkCIF report

## Figures and Tables

**Table 1:** Hydrogen-bond geometry (Å, °)

*D*—H⋯*A*	*D*—H	H⋯*A*	*D*⋯*A*	*D*—H⋯*A*
C15—H15⋯O1^i^	0.93	2.58	3.341 (2)	139
C7—H7⋯O2^ii^	0.93	2.70	3.340 (2)	126

Symmetry codes: (i) 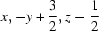; (ii) 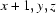.

**Fig. 1.**
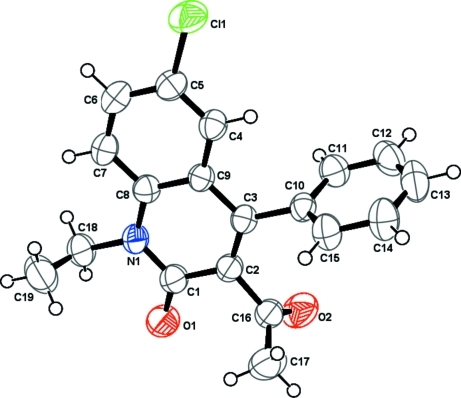
ORTEP diagram of molecule (I) with 50% probability displacement ellipsoids with atom labelling.

**Fig. 2.**
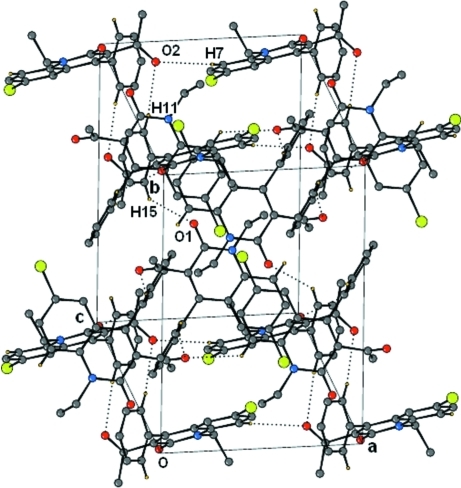
The crystal packing diagram of (I).The dotted lines indicate intermolecular C—H···O hydrogen bonds. All H atoms have been omitted for clarity.

## supplementary crystallographic information

### Comment

2-quinolinone is an important biosynthetic (Cobet *et al.*, 1971) and synthetic (Majumdar *et al.*, 2003; Yong *et al.*, 2001) precursor of quinoline alkaloids. Methylated compounds like 4-methoxy-1-methyl-2-quinolinone, folimine, 4,6-dimethoxy-1-methyl-2-quinolinone and 4,7,8-trimethoxy-1-methyl-2-quinolinone are widely distributed in nature (Nayar *et al.*, 1971; Lamberton *et al.*, 1953; Storer *et al.*, 1973). Due to the importance of these derivatives (Broadhurst *et al.*; 2001; Behrens, 1999; Broadhurst, 1991; Chao *et al.*, 1999; Kametani *et al.*, 1968) and in continuous of our interest in quinolines and isoquinolines (Choudhury & Guru Row 2006; Choudhury *et al.*, 2002; Hathwar *et al.*, 2008; Cho *et al.*, 2002; Manivel *et al.*, 2009) we report here crystal structure of the title compound.

All the bond lengths are within normal ranges in the title compound. The two carbonyl O atoms participate in intermolecular C—H···O hydrogen bonding resulting the close packing of the crystal structure in the unit cell (Figure 2).

### Experimental

The solution of 3-acetyl-6-chloro-4-phenylquinolin-2(1*H*)-one in DMF was treated with ethylbromide and K2CO3 taken in DMF and stirred at RT for 4hr. The reaction contents were poured in crushed ice and solid otanined was filtered, dried. Single-crystals were obtained by recrystallization from petrol ether and ethylacetate solvent mixture.

### Refinement

All  H atoms   were positioned geometrically and refined using a riding model with bond lengths C—H are 0.93 Å (for aromatic), 0.97 Å (for methylene) and 0.96 Å (for methyl). The *U*_iso_(H) = 1.5*U*_eq_(C) for methyl and *U*_iso_(H) = 1.2*U*_eq_(C) for all other carbon bound H atoms.

### Crystal data

**Table:**

C_19_H_16_ClNO_2_	*F*(000) = 680
*M**_r_* = 325.78	*D*_x_ = 1.315 Mg m^−^^3^
Monoclinic, *P*2_1_/*c*	Mo *K*α radiation, λ = 0.71073 Å
Hall symbol: -P 2ybc	Cell parameters from 1023 reflections
*a* = 9.6480 (8) Å	θ = 1.7–20.6°
*b* = 17.5756 (11) Å	µ = 0.24 mm^−^^1^
*c* = 9.9694 (7) Å	*T* = 290 K
β = 103.245 (8)°	Block, colorless
*V* = 1645.5 (2) Å^3^	0.21 × 0.16 × 0.15 mm
*Z* = 4	

### Data collection

**Table:**

Oxford Xcalibur Eos(Nova) CCD detector diffractometer	3061 independent reflections
Radiation source: Enhance (Mo) X-ray Source	1928 reflections with *I* > 2σ(*I*)
graphite	*R*_int_ = 0.052
ω scans	θ_max_ = 25.5°, θ_min_ = 3.1°
Absorption correction: multi-scan (*CrysAlis RED*; Oxford Diffraction, 2009)	*h* = −11→11
*T*_min_ = 0.925, *T*_max_ = 0.965	*k* = −21→21
21440 measured reflections	*l* = −12→12

### Refinement

**Table:**

Refinement on *F*^2^	Primary atom site location: structure-invariant direct methods
Least-squares matrix: full	Secondary atom site location: difference Fourier map
*R*[*F*^2^ > 2σ(*F*^2^)] = 0.040	Hydrogen site location: inferred from neighbouring sites
*wR*(*F*^2^) = 0.106	H-atom parameters constrained
*S* = 0.95	*w* = 1/[σ^2^(*F*_o_^2^) + (0.0592*P*)^2^] where *P* = (*F*_o_^2^ + 2*F*_c_^2^)/3
3061 reflections	(Δ/σ)_max_ = 0.001
210 parameters	Δρ_max_ = 0.16 e Å^−^^3^
0 restraints	Δρ_min_ = −0.27 e Å^−^^3^

### Special details

**Table:**

Geometry. All e.s.d.'s (except the e.s.d. in the dihedral angle between two l.s. planes) are estimated using the full covariance matrix. The cell e.s.d.'s are taken into account individually in the estimation of e.s.d.'s in distances, angles and torsion angles; correlations between e.s.d.'s in cell parameters are only used when they are defined by crystal symmetry. An approximate (isotropic) treatment of cell e.s.d.'s is used for estimating e.s.d.'s involving l.s. planes.
Refinement. Refinement of *F*^2^ against ALL reflections. The weighted *R*-factor *wR* and goodness of fit *S* are based on *F*^2^, conventional *R*-factors *R* are based on *F*, with *F* set to zero for negative *F*^2^. The threshold expression of *F*^2^ > σ(*F*^2^) is used only for calculating *R*-factors(gt) *etc*. and is not relevant to the choice of reflections for refinement. *R*-factors based on *F*^2^ are statistically about twice as large as those based on *F*, and *R*- factors based on ALL data will be even larger.

### Fractional atomic coordinates and isotropic or equivalent isotropic displacement parameters (Å^2^)

**Table:**

	*x*	*y*	*z*	*U*_iso_*/*U*_eq_	
Cl1	0.51092 (6)	1.05067 (3)	0.20536 (6)	0.0782 (2)	
N1	0.35571 (15)	0.79959 (8)	0.53674 (14)	0.0523 (4)	
O1	0.18412 (15)	0.72734 (8)	0.59753 (16)	0.0844 (5)	
O2	−0.11779 (16)	0.83560 (9)	0.49385 (18)	0.0873 (5)	
C1	0.2157 (2)	0.78011 (11)	0.52883 (19)	0.0562 (5)	
C2	0.10651 (18)	0.82606 (9)	0.43889 (18)	0.0488 (4)	
C3	0.13896 (17)	0.88600 (9)	0.36740 (17)	0.0448 (4)	
C4	0.32709 (19)	0.96195 (10)	0.29747 (17)	0.0502 (4)	
H4	0.2572	0.9916	0.2416	0.060*	
C5	0.46684 (19)	0.97641 (10)	0.30238 (18)	0.0527 (5)	
C6	0.5726 (2)	0.93221 (11)	0.38284 (19)	0.0585 (5)	
H6	0.6677	0.9419	0.3845	0.070*	
C7	0.53689 (19)	0.87387 (10)	0.46042 (18)	0.0554 (5)	
H7	0.6083	0.8443	0.5145	0.066*	
C8	0.39405 (18)	0.85855 (9)	0.45869 (17)	0.0467 (4)	
C9	0.28714 (17)	0.90307 (9)	0.37541 (16)	0.0434 (4)	
C10	0.02678 (17)	0.93280 (9)	0.27678 (17)	0.0460 (4)	
C11	−0.0010 (2)	1.00621 (10)	0.3121 (2)	0.0602 (5)	
H11	0.0483	1.0266	0.3956	0.072*	
C12	−0.1019 (2)	1.04958 (11)	0.2237 (2)	0.0705 (6)	
H12	−0.1209	1.0988	0.2485	0.085*	
C13	−0.1740 (2)	1.02063 (13)	0.1000 (2)	0.0743 (6)	
H13	−0.2407	1.0503	0.0402	0.089*	
C14	−0.1473 (2)	0.94779 (13)	0.0647 (2)	0.0736 (6)	
H14	−0.1972	0.9276	−0.0186	0.088*	
C15	−0.0470 (2)	0.90426 (11)	0.15166 (19)	0.0607 (5)	
H15	−0.0287	0.8551	0.1260	0.073*	
C16	−0.0442 (2)	0.80120 (11)	0.4330 (2)	0.0571 (5)	
C17	−0.0962 (2)	0.73258 (12)	0.3491 (2)	0.0816 (7)	
H17A	−0.1914	0.7210	0.3567	0.122*	
H17B	−0.0350	0.6903	0.3820	0.122*	
H17C	−0.0960	0.7422	0.2543	0.122*	
C18	0.4651 (2)	0.75412 (11)	0.6307 (2)	0.0636 (5)	
H18A	0.4254	0.7340	0.7044	0.076*	
H18B	0.5445	0.7868	0.6718	0.076*	
C19	0.5185 (3)	0.68935 (11)	0.5580 (3)	0.0872 (7)	
H19A	0.4404	0.6567	0.5174	0.131*	
H19B	0.5880	0.6609	0.6231	0.131*	
H19C	0.5613	0.7091	0.4873	0.131*	

### Atomic displacement parameters (Å^2^)

**Table:**

	*U*^11^	*U*^22^	*U*^33^	*U*^12^	*U*^13^	*U*^23^
Cl1	0.0683 (4)	0.0909 (4)	0.0817 (4)	−0.0115 (3)	0.0305 (3)	0.0206 (3)
N1	0.0456 (9)	0.0560 (9)	0.0506 (9)	0.0030 (7)	0.0012 (7)	0.0075 (7)
O1	0.0660 (10)	0.0864 (10)	0.0946 (11)	−0.0072 (8)	0.0056 (8)	0.0460 (9)
O2	0.0636 (10)	0.0908 (11)	0.1172 (13)	−0.0045 (8)	0.0406 (10)	−0.0133 (9)
C1	0.0508 (12)	0.0585 (12)	0.0568 (11)	−0.0002 (9)	0.0069 (9)	0.0104 (10)
C2	0.0433 (10)	0.0521 (10)	0.0504 (10)	−0.0007 (8)	0.0093 (8)	0.0018 (9)
C3	0.0434 (10)	0.0505 (10)	0.0398 (9)	0.0019 (8)	0.0081 (8)	0.0000 (8)
C4	0.0457 (11)	0.0591 (11)	0.0457 (10)	0.0028 (9)	0.0105 (8)	0.0031 (9)
C5	0.0511 (12)	0.0613 (11)	0.0480 (10)	−0.0042 (9)	0.0158 (9)	−0.0025 (9)
C6	0.0423 (11)	0.0728 (13)	0.0604 (12)	−0.0048 (10)	0.0115 (9)	−0.0070 (10)
C7	0.0426 (11)	0.0650 (12)	0.0545 (11)	0.0032 (9)	0.0030 (9)	−0.0006 (10)
C8	0.0463 (11)	0.0486 (10)	0.0435 (10)	−0.0001 (8)	0.0070 (8)	−0.0025 (8)
C9	0.0419 (10)	0.0467 (10)	0.0407 (9)	0.0003 (8)	0.0076 (8)	−0.0009 (8)
C10	0.0394 (10)	0.0529 (11)	0.0475 (10)	0.0041 (8)	0.0138 (8)	0.0063 (8)
C11	0.0623 (13)	0.0567 (12)	0.0617 (12)	0.0062 (10)	0.0146 (10)	−0.0015 (10)
C12	0.0735 (15)	0.0572 (12)	0.0872 (16)	0.0202 (11)	0.0316 (13)	0.0094 (12)
C13	0.0627 (14)	0.0896 (16)	0.0722 (15)	0.0289 (12)	0.0186 (12)	0.0254 (13)
C14	0.0675 (14)	0.0857 (15)	0.0602 (13)	0.0178 (12)	−0.0003 (11)	0.0037 (12)
C15	0.0607 (13)	0.0625 (12)	0.0547 (12)	0.0122 (10)	0.0045 (10)	0.0005 (10)
C16	0.0507 (12)	0.0596 (12)	0.0593 (12)	−0.0019 (10)	0.0093 (10)	0.0124 (10)
C17	0.0712 (15)	0.0829 (15)	0.0854 (16)	−0.0190 (12)	0.0067 (12)	−0.0039 (13)
C18	0.0598 (13)	0.0666 (13)	0.0579 (12)	0.0084 (10)	0.0002 (10)	0.0159 (10)
C19	0.0915 (17)	0.0672 (14)	0.1005 (18)	0.0208 (12)	0.0172 (14)	0.0104 (13)

### Geometric parameters (Å, °)

**Table:**

Cl1—C5	1.7344 (18)	C10—C15	1.381 (2)
N1—C1	1.378 (2)	C11—C12	1.383 (3)
N1—C8	1.396 (2)	C11—H11	0.9300
N1—C18	1.476 (2)	C12—C13	1.368 (3)
O1—C1	1.232 (2)	C12—H12	0.9300
O2—C16	1.198 (2)	C13—C14	1.368 (3)
C1—C2	1.460 (2)	C13—H13	0.9300
C2—C3	1.348 (2)	C14—C15	1.374 (3)
C2—C16	1.507 (2)	C14—H14	0.9300
C3—C9	1.445 (2)	C15—H15	0.9300
C3—C10	1.489 (2)	C16—C17	1.488 (3)
C4—C5	1.362 (2)	C17—H17A	0.9600
C4—C9	1.400 (2)	C17—H17B	0.9600
C4—H4	0.9300	C17—H17C	0.9600
C5—C6	1.383 (3)	C18—C19	1.502 (3)
C6—C7	1.375 (2)	C18—H18A	0.9700
C6—H6	0.9300	C18—H18B	0.9700
C7—C8	1.400 (2)	C19—H19A	0.9600
C7—H7	0.9300	C19—H19B	0.9600
C8—C9	1.405 (2)	C19—H19C	0.9600
C10—C11	1.380 (2)		
			
C1—N1—C8	122.28 (14)	C12—C11—H11	119.9
C1—N1—C18	116.83 (15)	C13—C12—C11	120.54 (19)
C8—N1—C18	120.89 (15)	C13—C12—H12	119.7
O1—C1—N1	121.27 (17)	C11—C12—H12	119.7
O1—C1—C2	121.48 (17)	C12—C13—C14	119.54 (19)
N1—C1—C2	117.22 (16)	C12—C13—H13	120.2
C3—C2—C1	122.30 (16)	C14—C13—H13	120.2
C3—C2—C16	123.02 (16)	C13—C14—C15	120.4 (2)
C1—C2—C16	114.68 (15)	C13—C14—H14	119.8
C2—C3—C9	118.67 (15)	C15—C14—H14	119.8
C2—C3—C10	121.86 (15)	C14—C15—C10	120.67 (18)
C9—C3—C10	119.42 (14)	C14—C15—H15	119.7
C5—C4—C9	120.94 (17)	C10—C15—H15	119.7
C5—C4—H4	119.5	O2—C16—C17	122.09 (19)
C9—C4—H4	119.5	O2—C16—C2	120.83 (18)
C4—C5—C6	120.58 (17)	C17—C16—C2	117.08 (18)
C4—C5—Cl1	119.19 (15)	C16—C17—H17A	109.5
C6—C5—Cl1	120.23 (14)	C16—C17—H17B	109.5
C7—C6—C5	119.91 (17)	H17A—C17—H17B	109.5
C7—C6—H6	120.0	C16—C17—H17C	109.5
C5—C6—H6	120.0	H17A—C17—H17C	109.5
C6—C7—C8	120.60 (17)	H17B—C17—H17C	109.5
C6—C7—H7	119.7	N1—C18—C19	112.27 (16)
C8—C7—H7	119.7	N1—C18—H18A	109.2
N1—C8—C7	121.44 (16)	C19—C18—H18A	109.2
N1—C8—C9	119.38 (15)	N1—C18—H18B	109.2
C7—C8—C9	119.17 (16)	C19—C18—H18B	109.2
C4—C9—C8	118.78 (16)	H18A—C18—H18B	107.9
C4—C9—C3	121.17 (15)	C18—C19—H19A	109.5
C8—C9—C3	120.01 (15)	C18—C19—H19B	109.5
C11—C10—C15	118.71 (16)	H19A—C19—H19B	109.5
C11—C10—C3	121.22 (16)	C18—C19—H19C	109.5
C15—C10—C3	120.00 (15)	H19A—C19—H19C	109.5
C10—C11—C12	120.13 (18)	H19B—C19—H19C	109.5
C10—C11—H11	119.9		
			
C8—N1—C1—O1	179.22 (17)	C7—C8—C9—C4	−0.6 (2)
C18—N1—C1—O1	0.2 (3)	N1—C8—C9—C3	−2.1 (2)
C8—N1—C1—C2	−2.6 (2)	C7—C8—C9—C3	177.21 (15)
C18—N1—C1—C2	178.42 (15)	C2—C3—C9—C4	176.41 (16)
O1—C1—C2—C3	177.17 (18)	C10—C3—C9—C4	−1.2 (2)
N1—C1—C2—C3	−1.0 (3)	C2—C3—C9—C8	−1.3 (2)
O1—C1—C2—C16	−2.7 (3)	C10—C3—C9—C8	−178.92 (15)
N1—C1—C2—C16	179.10 (16)	C2—C3—C10—C11	109.3 (2)
C1—C2—C3—C9	2.9 (2)	C9—C3—C10—C11	−73.2 (2)
C16—C2—C3—C9	−177.26 (15)	C2—C3—C10—C15	−73.9 (2)
C1—C2—C3—C10	−179.55 (16)	C9—C3—C10—C15	103.69 (19)
C16—C2—C3—C10	0.3 (3)	C15—C10—C11—C12	0.6 (3)
C9—C4—C5—C6	1.2 (3)	C3—C10—C11—C12	177.48 (16)
C9—C4—C5—Cl1	−179.29 (13)	C10—C11—C12—C13	−0.7 (3)
C4—C5—C6—C7	−1.0 (3)	C11—C12—C13—C14	1.0 (3)
Cl1—C5—C6—C7	179.43 (13)	C12—C13—C14—C15	−1.1 (3)
C5—C6—C7—C8	0.1 (3)	C13—C14—C15—C10	1.0 (3)
C1—N1—C8—C7	−175.20 (16)	C11—C10—C15—C14	−0.7 (3)
C18—N1—C8—C7	3.8 (2)	C3—C10—C15—C14	−177.66 (17)
C1—N1—C8—C9	4.1 (2)	C3—C2—C16—O2	−76.4 (2)
C18—N1—C8—C9	−176.91 (16)	C1—C2—C16—O2	103.5 (2)
C6—C7—C8—N1	−179.99 (15)	C3—C2—C16—C17	103.7 (2)
C6—C7—C8—C9	0.7 (3)	C1—C2—C16—C17	−76.4 (2)
C5—C4—C9—C8	−0.4 (2)	C1—N1—C18—C19	93.7 (2)
C5—C4—C9—C3	−178.10 (16)	C8—N1—C18—C19	−85.3 (2)
N1—C8—C9—C4	−179.89 (14)		

### Hydrogen-bond geometry (Å, °)

**Table:**

*D*—H···*A*	*D*—H	H···*A*	*D*···*A*	*D*—H···*A*
C15—H15···O1^i^	0.93	2.58	3.341 (2)	139
C7—H7···O2^ii^	0.93	2.70	3.340 (2)	126

Symmetry codes: (i) *x*, −*y*+3/2, *z*−1/2; (ii) *x*+1, *y*, *z*.
